# Psychometric Properties of Satisfaction with Life Scale (SWLS) and Psychological Capital Questionnaire (PCQ-24) in the Lithuanian Population

**DOI:** 10.3390/ijerph18052608

**Published:** 2021-03-05

**Authors:** Aiste Dirzyte, Aidas Perminas, Egle Biliuniene

**Affiliations:** 1 Faculty of Creative Industries, Vilnius Gediminas Technical University, Traku Str. 1, 01132 Vilnius, Lithuania; 2 Institute of Psychology, Mykolas Romeris University, Ateities Str. 20, 08303 Vilnius, Lithuania; egle.sabaityte@gmail.com; 3 Department of Psychology, Vytautas Magnus University, K. Donelaičio Str. 58, 44248 Kaunas, Lithuania; aidas.perminas@vdu.lt

**Keywords:** psychological capital, PsyCap, satisfaction with life, self-efficacy, hope, optimism, resilience, Lithuania

## Abstract

This study aimed to explore psychometric properties of satisfaction with life scale (SWLS) and psychological capital questionnaire (PCQ-24) in the Lithuanian representative sample (*n* = 2003, M = 50.67, SD = 17.46). It was significant to validate instruments concerning the fact that Lithuanians’ life satisfaction surveys demonstrated divergent results depending on the assessment tools they used. This study applied the SWLS, created by Diener et al. (1985), and the PCQ-24, created by Luthans et al. (2007). The findings demonstrated the internal consistency of the SWLS instrument, evidencing it as an adequate measure to evaluate satisfaction with life (α = 0.893; TLI = 0.988; NFI = 0.997; RMSEA = 0.059 [0.033–0.088]; CFI = 0.998; SRMR = 0.0077; AVE = 0.764; CR = 0.886). The Lith-PCQ-21 analysis demonstrated the internal consistency of the instrument (α = 0.957) and good fit of the factorial structure (χ^2^ = 2305.383; DF = 185; TLI = 0.915; NFI = 0.920; RMSEA = 0.077 [0.075–0.080]; CFI = 0.925; SRMR = 0.0450; AVE = 0.814; CR = 0.946), evidencing the instrument as an adequate measure to evaluate psychological capital. This research confirmed that both instruments (SWLS and Lith-PCQ-21) not only have an acceptable validity, including construct validity, but they are also interrelated (χ^2^ = 3088.762; DF = 294; TLI = 0.913; NFI = 0.914; RMSEA = 0.070 [0.068–0.073]; CFI = 0.922; SRMR = 0.0469), and can be considered appropriate for monitoring life satisfaction and psychological capital of the Lithuanian population.

## 1. Introduction

Life satisfaction represents an appraisal of one’s life as a whole and is the cognitive component of the broader construct of subjective wellbeing [1]. Research shows that high life satisfaction correlates with better health [2,3], the absence of difficulties such as depression [4,5], or sleep disturbances [6]. Furthermore, individuals who are satisfied with life are good problem solvers and tend to be more resistant to stress [7,8,9].

A literature search in the Social Sciences Citation Index of the Web of Science Core Collection (on 2 January 2021) found 7214 published articles mentioning in the title “life satisfaction” and around 65,000 articles mentioning the topic “life satisfaction”, which proves that life satisfaction is a highly researched construct. Research suggests that people can answer questions regarding their life satisfaction reliably and that these data have acceptable validity, including construct validity [1,9,10,11,12]. Research indicates that attempts to compare absolute levels of life satisfaction across nations have proven problematic due to cultural differences [13], but presumes there is good agreement about the efficiency of subjective life satisfaction as an intra-nation measure [14].

Based on different surveys (Eurostat, Eurobarometer) on life satisfaction in the same country, it is evident that different studies might report contradictory estimates of life satisfaction in the same state depending on the assessment tools they use. Life satisfaction surveys conducted in Lithuania show that different studies demonstrate dissimilar results. Given that there are many suicides in Lithuania and that suicides are negatively related to life satisfaction [15], it is essential to validate reliable instruments to study the Lithuanian population. 

The current population of Lithuania, a country in the Baltic region of Europe, is 2,700,200, based on Worldometer elaboration of the latest United Nations data and is equivalent to 0.03% of the total world population. The population density in Lithuania is 43 per km^2^ (112 people per mi^2^), the total land area is 62,674 km^2^ (24,199 sq. miles). 71.3% of the population is urban, the median age in Lithuania is 45.1 years [16].

**Figure 1:**
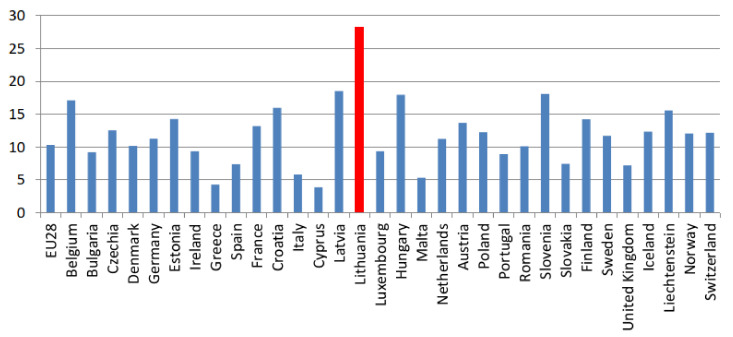
Total suicides per 100,000 inhabitants. Source: Eurostat, standardized death rate per 100,000 inhabitants, the year 2016. The total refers to both the male and female population.

For several decades, suicide rates in Lithuania have been higher than in other EU countries [17]. As shown in Figure 1, Lithuania was a clear outlier in the EU in 2016. Similarly, in 2017, Lithuania registered the highest rate of suicide at 26 deaths per 100,000 inhabitants among the European states. 

Research indicates (Table 1) there is a strong negative correlation between life satisfaction and suicides in Lithuania (r = −0.644, *p* < 0.001), while suicides are also related to unemployment [15]. 

However, there is not enough evidence suggesting a causal relationship between suicides in Lithuania and unemployment. Moreover, the Lithuanian population’s health or socio-economic data point to a number of other probable risk factors. 

**Figure 2:**
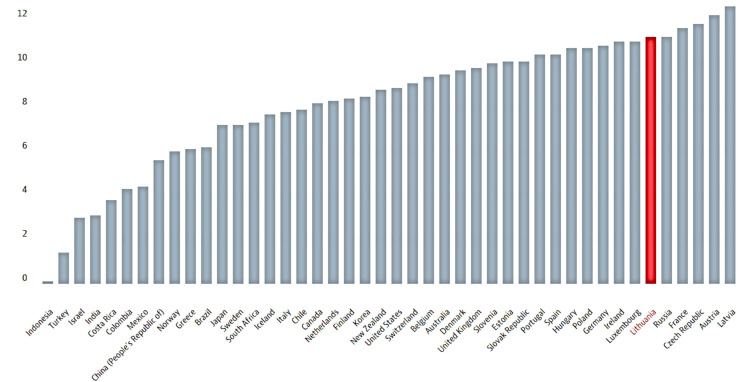
Alcohol consumption in 2018. Total, Liters/capita (aged 15 and above). Reprinted with permission from ref. [19]. Copyright 2017 Mykolas Romeris University.

In 2017, the Lithuanian people aged 15 and above drank on average 12.3 L of pure ethanol per year, while the least drinking nations were Indonesia, Turkey, and Israel, with 0.3, 1.4 and 2.6 L, respectively [18]. In 2018, the Lithuanian people drank on average 11.2 L of pure ethanol (Figure 2), which is a significant decrease from 14.7 L per year in 2014–2015 [18]. 

Moreover, Lithuania is still suffering high socio-economic inequality [19]. The study on Life Quality, conducted in Lithuania in 2014, demonstrated strong associations between the net income per month and overall satisfaction with life [20]. Some years later, life satisfaction in Lithuania was found to be significantly related to objective and subjective socioeconomic status [21] and emotional experiences during the past four weeks [22].

**Figure 3:**
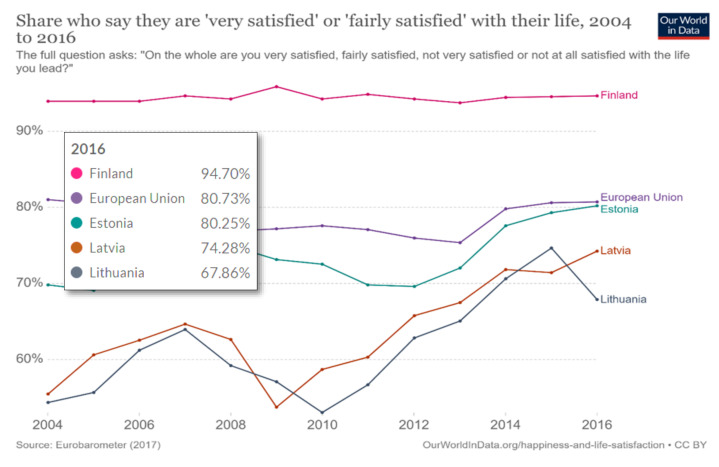
Percentage of people “very satisfied” or “fairly satisfied” with their lives in several countries.

As shown in Figure 3, Eurobarometer revealed that Lithuanians have been much more unsatisfied with their lives than their neighbors Latvians and Estonians for several years. However, life satisfaction in Lithuania has obviously increased after joining the European Union (EU) in 2004 and decreased after adopting the Euro currency on 1 January 2015. 

**Figure 4:**
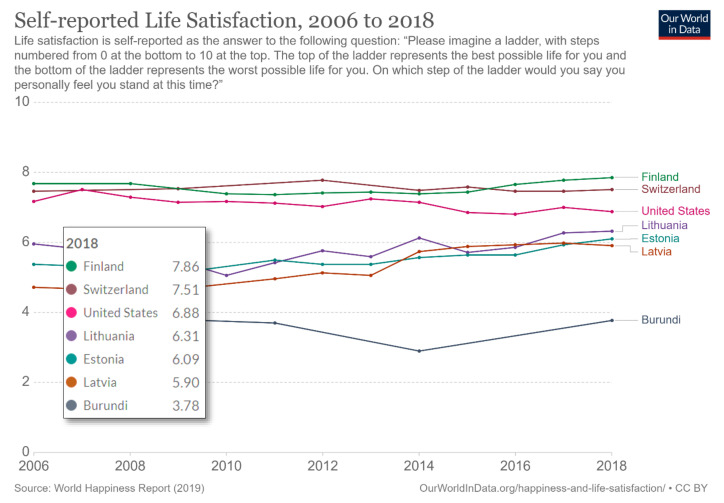
Self-reported life satisfaction estimates.

However, as shown in Figure 4, the World Happiness report discovered that Lithuanians have been more satisfied with life than Estonians or Latvians. This report also showed that the most satisfied with life were people in Finland, and the least satisfied with life were people in Burundi [23].

Besides, in 2019, the OECD Better Life Index, created in 2011 by the Organization for Economic Co-operation and Development, which measures life satisfaction by asking how people evaluate their life as a whole rather than their current feelings on a scale from 0 to 10, showed that when asked to rate their general satisfaction with life Lithuanians gave it a 5.9 grade on average, lower than the OECD average of 6.5 [18]. 

Thus, it is evident that national life satisfaction surveys need to apply measurement tools that provide replicable, stable, and reliable results. 

While being concerned about the problems of suicides and life satisfaction levels in Lithuania, this study applies a positive psychology framework signifying the importance of accurate evaluation, promoting, and consolidation of both people’s individual and social resources and strengths to improve psychological wellbeing at individual, national, and cross-national levels [24]. 

Research indicates that satisfaction with life scale (SWLS), developed by E. Diener et al. (1985) [10], have favorable psychometric properties, including high internal consistency and reliability, and this scale has constantly been used for life satisfaction measurement in several countries [25]. 

The SWLS is exclusively focused on assessing global life satisfaction and does not tap related constructs such as positive emotions. Scores on the SWLS correlate with other measures of subjective wellbeing and specific personality characteristics [26]. Therefore, after validation, it would be appropriate to use this tool as an intra-nation measure to monitor the Lithuanian population’s life satisfaction. 

Furthermore, as mentioned above, Lithuania is facing problems of a relatively low life satisfaction, suicides, and unemployment. More than a decade ago, Fred Luthans and colleagues introduced the construct of psychological capital (PsyCap) and psychological capital intervention. Studies have confirmed that psychological capital and its interventions can effectively enhance employability [27], or at least they are related to reduced work-related problems [28,29,30,31,32,33,34,35] and increase in life satisfaction [36,37]. In the context of the Lithuanian situation, the accurate assessment of psychological capital acquires a special significance. 

A literature search in the Social Sciences Citation Index of the Web of Science Core Collection (on 2 January 2021) found 868 articles mentioning in the title “psychological capital” and 353 articles mentioning the topic “PsyCap”.

A construct of psychological capital emerged around a decade ago and was defined as “individual’s positive state of development […], characterized by (1) having confidence (self-efficacy) to take on and put in the necessary effort to succeed at challenging tasks; (2) making positive attributions (optimism) about succeeding now and in the future; (3) persevering toward goals and, when necessary, redirecting paths to goals (hope) to succeed; and (4) when beset by problems and adversity, sustaining and bouncing back and even beyond (resilience) to attain success” [38] (p. 3). In other words, psychological capital refers to a constructive evaluation of one’s ability to handle challenges with sustained effort, and this appraisal reflects four dimensions: self-efficacy, hope, resilience, and optimism.

As mentioned above, research has documented associations between psychological capital and several wellbeing outcomes, including health [37,38,39,40,41,42], life satisfaction [36,43], quality of life [44], employment [27], academic performance [45], not to mention many positive work-related outcomes [33,46,47,48,49]. 

The capacity to persevere and overcome emotional difficulties may be critical in the Lithuanian context, which evidences the high rates of unsuccessful struggles with emotional burdens. Research shows that individuals higher on PsyCap are more likely to engage in opportunities to sustain and improve wellbeing and more likely to persist in efforts to achieve their goals [50]. Besides, positive experiences are likely to set constructive progress whereby people see themselves as more capable of taking on more significant challenges with each success [51]. Furthermore, a recent meta-analysis by Donaldson, S. I., Chan, L. B., Villalobos, J., & Chen, C. L. suggests that interventions that target and improve hope, efficacy, resilience, and optimism (psychological capital) can be highly effective at improving wellbeing and positive functioning at work [52]. 

Luthans, Avolio, Avey, and Norman in 2007 developed the Psychological Capital Questionnaire (PCQ-24), which is the main instrument to measure the construct of psychological capital [38]. The PCQ-24 was based on four published measures on self-efficacy [53], hope [54], optimism [55], and resilience [56]. 

In 2012, to help minimize problems related to social desirability, Harms and Luthans (2012) developed the Implicit Psychological Capital Questionnaire (I-PCQ), which demonstrated acceptable structural validity, was resistant to response distortion, and predicted work outcomes above and beyond the widely used self-report PCQ and Big Five personality traits [57]. 

In 2016, Lorenz, Beer, Pütz and Heinitz (2016) [58], based on research that PsyCap is shown to be linked to outcomes of general importance for individuals [59], designed and validated a universal measure for the PsyCap construct (CPC-12), with applications for all domains of life, not only the organizational context. 

During the last decade, the PCQ-24 itself and its versions (PCQ-12, I-PCQ, CPC-12, and others) were adapted to contexts other than the workplace, including health [42], relationships [60], sports [32], education [61]. 

In 2013, Dawkins et al. (2013) made a systematic review of 29 psychometric studies using the different PCQ versions [62]. The results of the exploratory and confirmatory analyzes (for both the PCQ-24 and PCQ-12) demonstrated significant reliability (internal consistency higher than 0.70) in different cultural and organizational contexts, indicating the potential of the instruments to measure the PsyCap reliably.

During the last decade, the PCQ-24 has been validated in many cultural contexts and countries, such as the USA [38], South Africa [63,64], China [65], Portugal [66], Italy [67], France [68], Brasilia [69], Pakistan [70], among others. Most of the studies confirmed the original structure of the PsyCap construct, with the distribution in four factors, with Cronbach’s alphas between 0.70 and 0.95. 

Nonetheless, several studies indicated that negatively worded items of the PCQ-24 tend to be problematic [62,71,72,73], and some researchers suggested eliminating them from the final solutions [69]. 

Despite the concerns with negatively worded items, most of previous research indicated that Psychological Capital Questionnaire (PCQ-24), developed by F. Luthans et al., has acceptable construct validity [38]. Thus, we assumed that it would be appropriate to use this tool in Lithuania after validation. 

Furthermore, positive psychology is an umbrella term for models and exploration about what makes life most worth living [74]. Even though positive judgments about life such as life satisfaction or positive resources such as psychological capital are among the central concerns of positive psychology, no studies were conducted in the Lithuanian population to evaluate psychometric properties of the SWLS and the PCQ-24, which are the leading measures to evaluate satisfaction with life and psychological capital.

As mentioned above, this study applies a positive psychology framework focused on an approach based on keywords such as flourishing, growth, flow, enrichment, flexible change, and suggesting significance of precise evaluation and consolidation of individual and social resources and strengths [75] to improve psychological wellbeing [24], including emotional, social, and existential well-being [76] at individual, national, or cross-national levels.

Therefore, this study aimed to examine the reliability and validity of the PsyCap Questionnaire (PCQ-24) and the Satisfaction with Life Scale (SWLS). The adaptation of the instruments in Lithuania is conducive to add to cross-cultural research and benchmarks of well-being research across countries.

Based on previous research on psychological capital and satisfaction with life, we hypothesized (1) the existence of a common higher-order PsyCap factor that is superordinate and represents the four sub-dimensions: resilience, self-efficacy, hope, optimism, and targeted to verify it by confirmatory factor analysis. We also hypothesized (2) the existence of a life satisfaction factor and aimed to explore its association with psychological capital. We hypothesized that (3) the Lithuanian versions of PCQ-24 and SWLS will demonstrate satisfactory reliability and validity.

Furthermore, some research indicated gender differences in life satisfaction [77] and psychological capital [78] in different countries. Additionally, previous research suggested the possible impact of rural and urban factors on satisfaction with life [79] and psychological capital [80], and some studies suggested the possible impact of age on satisfaction with life [81] and psychological capital [82]. Therefore, along with the instruments’ validation, we also intended to analyze satisfaction with life and psychological capital evidence related to sex, age, and living area in the Lithuanian population.

## 2. Materials and Methods

### 2.1. Sample

This study used a test design utilizing a heterogeneous random sample of 2003 persons representing the Lithuanian population. All the participants were personally asked to participate in the study and were personally interviewed at their homes. The participants were selected and interviewed by the professionals of the Lithuanian sociological research company, which ensured that the sample was representative to the population and collected the data in Vilnius, Kaunas, Klaipėda, Šiauliai, Panevėžys, Druskininkai, Kretinga, Alytus, Šakiai, Pakruojis, Utena, Tauragė, Švenčionys, Raseiniai, Kupiškis, Akmenė, Rokiškis, Lazdijai, Telšiai, Mažeikiai, Marijampolė, Anykščiai, Varėna, Molėtai and Ukmergė districts, overall in 20 cities and 29 villages. Participants were collected from the database in a multi-scaled probabilistic way so that every citizen of Lithuania might have an equal probability to be interviewed and were personally asked to participate in the study. Upon agreement, the interviewers arrived at the participants’ home and informed the participants about the purpose of the study. The participants were also informed, that their personal data (names, etc.) are omitted in the questionnaire. The interviewers did not mark personal data in the questionnaire to ensure confidentiality and anonymity; thus, the research team received anonymous data of the sample. The interviewers were present during the compilation and the questions were asked directly by the interviewer. 

The study’s Lithuanian subjects included 46.8 percent of men (95% CI = 44.7, 49.0) and 53.2 percent of women (95% CI = 51.0, 55.3). The respondents’ mean age was 50.67 years (SD = 17.46, 95% CI = 49.90, 51.43, age range = 18 to 90 years). 71.7% of respondents lived in urban area, and 28.3% (95% CI = 26.3, 30.3) lived in rural area. Most of the participants were married (48.6 percent, 95% CI = 46.3, 50.7), some were widows (15 percent, 95% CI = 13.4, 16.6), some indicated that they lived alone (13.3 percent, 95% CI = 11.9, 14.8), they are separated (12.8 percent, 95% CI = 11.3, 14.4), or lived with a partner (8.9 percent, 95% CI = 7.7, 10.2). 

Participation in the study was anonymous and voluntary, and the participants did not receive any compensation. This study’s data were taken from a more extensive study on Lithuanians quality of life that was given ethical approval by the ethics committee at the Quality-of-Life Laboratory, Mykolas Romeris University (VP1-3.1-ŠMM-07-K-03-032, 2017-1-LT01-KA201-035296), and that was conducted following the Declaration of Helsinki.

### 2.2. Instruments

This study used two instruments, the translated Lithuanian version of the Satisfaction with Life Scale (SWLS) and the translated Lithuanian version of the Psychological Capital Questionnaire—PCQ-24. The permission to use PCQ-24 for research purposes was given to Aiste Dirzyte by Fred Luthans in 2013. The SWLS is free to use without permission for research purposes, as indicated by the scale’s authors (Ed Diener, Robert A. Emmons, Randy J. Larsen, and Sharon Griffin, 1985, an article in the Journal of Personality Assessment) [10]. To make sure that the Lithuanian items correspond as closely as possible to the English items, the original items of both instruments were translated into Lithuanian and back translated [83] by Aiste Dirzyte, Egle Biliuniene, and Aidas Perminas.

#### 2.2.1. The SWLS

The SWLS is a short 5-item instrument designed to measure global cognitive judgments of satisfaction with one’s life. We applied the Satisfaction with Life Scale (SWLS) of E. Diener and colleagues [10] to examine psychometric properties of the scale in the Lithuanian population. The response pattern followed a 7-point Likert scale ranging from 7 (totally agree) to 1 (totally disagree). The results presented below indicates that the Lithuanian version of the SWLS demonstrated high internal consistency, reliability, and validity.

#### 2.2.2. The PCQ-24

We applied the PCQ-24 scale [38] to assess respondents’ positive psychological capital. Psychological Capital or PsyCap is a higher-order construct consisting of four subscales, each comprised of six items. The subscales include hope, efficacy, resilience, and optimism. Some sample items for PsyCap are the following: “I feel confident analyzing a long-term problem to find a solution” (Efficacy subscale); “There are lots of ways around my problem” (Hope subscale); “I always look on the bright side of things” (Optimism scale); and “I usually manage difficulties one way or another” (Resilience scale). In this study, the response pattern followed a 6-point Likert scale ranging from 6 (totally agree) to 1 (totally disagree). The results presented below indicates that the Lithuanian version of the PCQ-24 demonstrated high internal consistency, reliability, and validity, even though minor inconsistencies were observed, possibly due to cultural differences.

### 2.3. Statistical Analyses

In the full sample of 2003 participants, a total of 1979 (98.8%) participants had no missing data. As the number of cases with missing values was small (*n* = 24; 1.2%), we used listwise deletion of cases with missing values. Therefore, all analyses were conducted using a sample of 1979 individuals (47.2% of males, 52.8% of females) with no missing data. 

For data analysis, we used SPSS v.26.0. The structural equation modeling (SEM), confirmatory factor analysis (CFA) models for the PCQ-24 (four-factors), and the SWLS (single-factor) were conducted using AMOS v.26.0. Model fit was evaluated based on the CFI (Comparative Fit Index), the Normed Fit Index (NFI), the Tucker–Lewis coefficient (TLI), RMSEA (Root Mean Square Error of Approximation), and SRMR (Standardized Root Mean Square Residual), whereas the χ^2^ was used for descriptive purposes only because it is susceptible to sample size [84]. The values higher than 0.90 for CFI, NFI, TLI, and values lower than 0.08 for RMSEA and SRMR were considered as indicative of a good fit [85]. In this research, we considered *p*-values less than 0.01 to be statistically significant [86].

The Shapiro–Wilk test showed the departure from normality for the variables of life satisfaction, W (1978) = 0.994, *p* < 0.001; psychological capital, W (1978) = 0.993, *p* < 0.001; self-efficacy, W (1978) = 0.981, *p* < 0.001; hope, W (1978) = 0.992, *p* < 0.001; resilience, W (1978) = 0.988, *p* < 0.001, and optimism W (1978) = 0.977, *p* < 0.001. Similarly, Kolmogorov–Smirnov test showed that data were non-normally distributed for the variables of life satisfaction, D (1978) = 0.061, *p* < 0.001; psychological capital, D (1978) = 0.038, *p* < 0.001; self-efficacy, D (1978) = 0.075, *p* < 0.001; hope, D (1978) = 0.062, *p* < 0.001; resilience, D (1978) = 0.082, *p* < 0.001, and optimism D (1978) = 0.094, *p* < 0.001. 

The distribution was moderately skewed: life satisfaction skewness = −0.001 (SE = 0.056), kurtosis = −0.174 (SE = 0.112); psychological capital skewness= −0.281 (SE = 0.056), kurtosis = 0.180 (SE = 0.112); self-efficacy skewness = −0.418 (SE = 0.056), kurtosis = 0.279 (SE = 0.112); hope skewness= −0.222 (SE = 0.056), kurtosis = −0.055 (SE = 0.112); resilience skewness = −0.257 (SE = 0.056), kurtosis = 0.301 (SE = 0.112); optimism skewness = −0.408 (SE = 0.056), kurtosis = 0.521 (SE = 0.112).

Therefore, we conducted a square root transformation (SQRT) of significantly negatively skewed variables to create normally distributed variables and conduct the CFA analyses.

Furthermore, composite reliability (CR) was used as measure of internal consistency of the factors, where values greater 0.70 indicate good reliability. For convergent validity, average variance extracted (AVE) had to be equal or greater than 0.50 and lower than CR, and for discriminant validity, SQRT(AVE) had to be higher than the correlations between items [87].

## 3. Results

Means, standard deviations, and correlations between SWLS items in the Lithuanian population are reported in Table 2. 

Means, standard deviations, and correlations between psychological capital’s sub-dimension self-efficacy items in the Lithuanian population are reported in Table 3. 

Means, standard deviations, and correlations between psychological capital’s sub-dimension hope items in the Lithuanian population are reported in Table 4.

Means, standard deviations, and correlations between psychological capital’s sub-dimension resilience items in the Lithuanian population are reported in Table 5. 

Means, standard deviations, and correlations between psychological capital’s sub-dimension optimism items in the Lithuanian population are reported in Table 6. 

Furthermore, the factorability of the SWLS and the PCQ-24 items was examined. The correlation analysis suggested that the SWLS Items correlated well (at least 0.5 with one another), suggesting reasonable factorability, but some of the PCQ-24 Items correlated below 0.3, suggesting not acceptable factorability. 

Exploratory factor analysis of the SWLS confirmed a single factor structure. The Kaiser-Meyer-Olkin measure of sampling adequacy for the SWLS was 0.865, above the commonly recommended value of 0.6, and the Bartlett’s test of sphericity was significant (χ^2^ (10) = 5566.892, *p* < 0.001), and the communalities were all above 0.3 (SWLS Item1 = 0.544; SWLS Item2 = 0.614; SWLS Item3 = 0.675; SWLS Item4 = 0.594, SWLS Item5 = 0.459), confirming that each item shared some common variance with other items.

**Figure 5:**
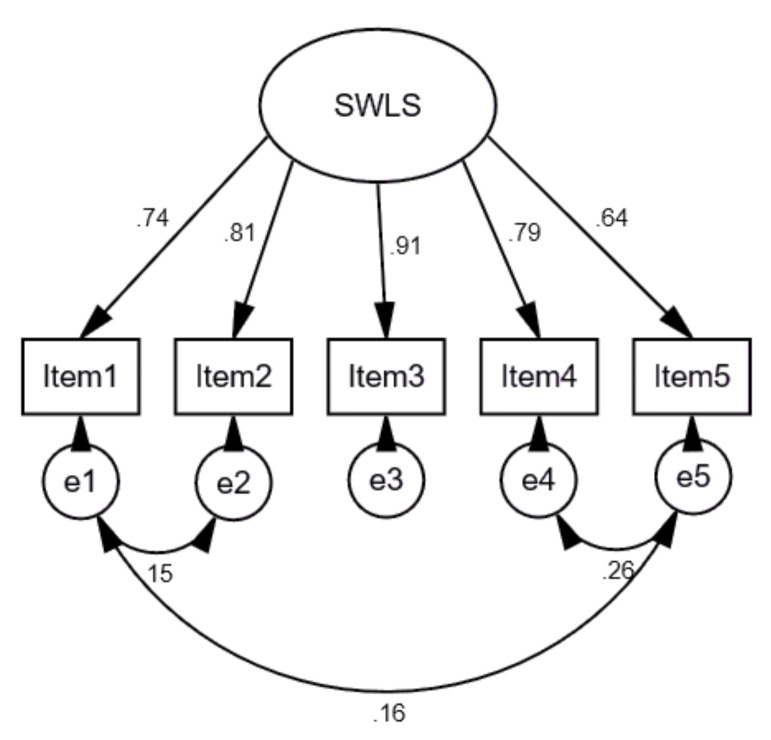
Standardized results of the SWLS items. χ^2^ = 15.157; DF = 2; TLI = 0.988; NFI = 0.997; RMSEA = 0.059 [0.033–0.088]; CFI = 0.998; SRMR = 0.0077.

Afterward, we conducted a CFA analysis for the SWLS data on the Lithuanian representative sample. Standardized results are presented in Figure 5. We freed residual covariances of some of the items in the SWLS in order to transparently demonstrate that some of the Items (1 and 2, 3 and 4, 1 and 4) share common content, and there might be a methodological similarity across the items. 

As mentioned above, to assess the model fit, the Comparative Fit Index (CFI), the Normed Fit Index (NFI), the Tucker–Lewis coefficient (TLI), Root Mean Square Error of Approximation (RMSEA), and SRMR (Standardized Root Mean Square Residual) were used. Findings revealed that the fit of the model was good, χ^2^ = 15.157; DF = 2; TLI = 0.988; NFI = 0.997; RMSEA = 0.059 [0.033–0.088]; CFI = 0.998; SRMR = 0.0077. 

However, as the most important CFA assumption is that errors must be uncorrelated, we have also tested a concurring model, without residual covariances between Items 1 and 2, 3 and 4, 1 and 4. The comparison of the models is presented in Table 7. The results suggest that the SWLS demonstrates a single factor structure and indicates that the reliability and the validity of both models is good (Composite reliability is >0.8, and Average Variance Extracted is >0.7, and Discriminant Validity scores were all higher than the correlations between items), but the FIT indices are better for the model with the correlated errors.

Furthermore, we examined the factorability of the PCQ-24 items. As mentioned above, correlational analysis of some items suggested not satisfactory factorability. 

Exploratory factor analysis of the translated version of the PCQ-24 indicated a five-factor structure, which was not in line with the propositions of the authors [38]. The Kaiser-Meyer-Olkin measure of sampling adequacy for the PCQ-24 was 0.963, above the commonly recommended value of 0.6, and the Bartlett’s test of sphericity was significant (χ^2^ (276) = 29,640.890, *p* < 0.001), but some of the communalities (Unidentified factor) were below 0.3, suggesting the necessity to exclude these items (23, 20, 13) from the factorial structure of PCQ-24. The results of the exploratory factor analysis for Lithuanian version of the PCQ-24 items are presented in Table 8, which displays the items and rotated factor loadings, with other factor loadings omitted to improve clarity.

**Figure 6:**
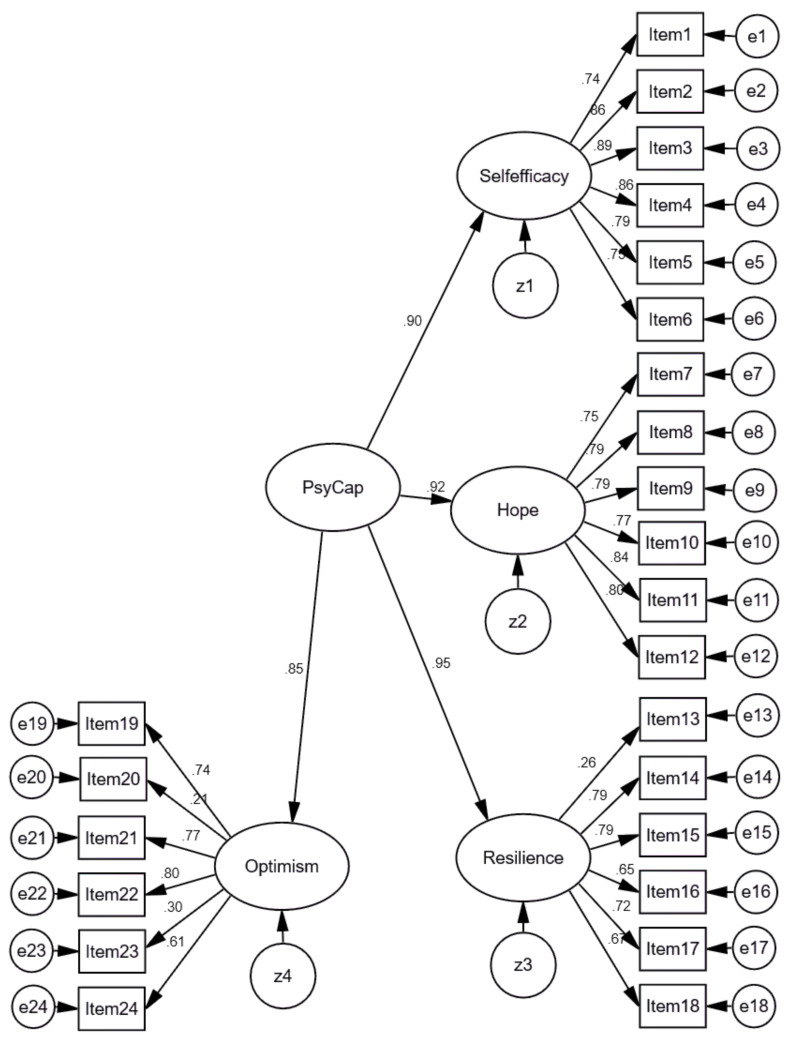
Standardized results of Model 1 of the PCQ-24 with 24 items. χ^2^ = 3079.712; DF = 248; TLI = 0.893; NFI = 0.897; RMSEA = 0.077 [0.075–0.080]; CFI = 0.904; SRMR = 0.0528.

As the Exploratory Factor Analysis did not confirm the factor structure proposed by Luthans et al. (2007) [38], and Item 7 was assigned to the self-efficacy factor, we conducted the Confirmatory Factor analysis with the 24 items, based on the propositions of the authors of the PCQ-24. Standardized results of the model with 24 items are presented in Figure 6. As mentioned above, to assess the model fit, the Comparative Fit Index (CFI), the Normed Fit Index (NFI), the Tucker–Lewis coefficient (TLI), Root Mean Square Error of Approximation (RMSEA), and SRMR (Standardized Root Mean Square Residual) were used. Findings revealed that the fit of the original 24 items model was acceptable, χ^2^ = 3079.712; DF = 248; TLI = 0.893; NFI = 0.897; RMSEA = 0.077 [0.075–0.080]; CFI = 0.904, Standardized RMR = 0.0528. 

However, as seen in Figure 6, the three (negative) inverted items had factor loads below 0.45 (0.26 for Item 13, 0.21 for Item 20, and 0.30 for Item 23), while the minimum value required for confirmatory factor analyzes must be ≥0.45, according to Tabachnick and Fidell (2018) [88].

Therefore, as the EFA and the CFA suggested that items 13, 20 and 23 did not contribute to the four-factor structure of the PCQ-24 and just worsened it, we have excluded the three inverted items (13, 20, and 23), and the PCQ-24 scale was shortened to 21 items (self-efficacy = 6 items, hope = 6 items, resilience = 5 items, and optimism = 4 items). 

Exploratory factor analysis of the translated version of the PCQ-21 indicated a four-factor structure, which was in line with the propositions of the authors [38]. The Kaiser-Meyer-Olkin measure of sampling adequacy for the PCQ-21 was 0.965, above the commonly recommended value of 0.6, and the Bartlett’s test of sphericity was significant (χ^2^ (210) = 28,546.089, *p* < 0.001), and the communalities were all above 0.3, confirming that each item shared some common variance with other items. Table 9 displays the items and rotated factor loadings for the Lithuanian version of the PCQ-21 items, with other factor loadings omitted to improve clarity.

**Figure 7:**
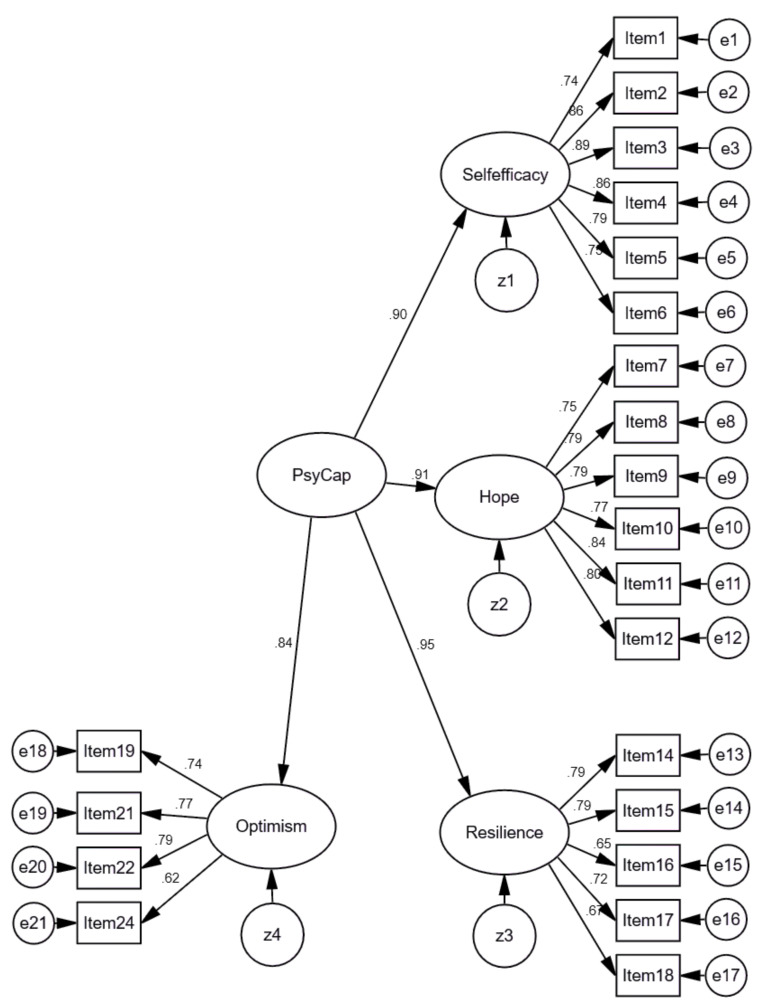
Standardized results of Model 2 of the PCQ-24 with 21 items. χ^2^ = 2305.383; DF = 185; TLI = 0.915; NFI = 0.920; RMSEA = 0.077 [0.075–0.080]; CFI = 0.925; SRMR = 0.0450.

However, the Exploratory Factor Analysis did not confirm the model proposed by Luthans et al. (2007) [38] perfectly, as Item 7 again was assigned to the self-efficacy factor. Therefore, we created a CFA model of the PCQ-24 with 21 items, but following the structure, proposed by the PCQ-24 authors [34]. This second-order model’s confirmatory factor analysis revealed a higher fit than a CFA model with 24 items. Standardized results of the model are presented in Figure 7. In order to assess the model fit, the Comparative Fit Index (CFI), the Normed Fit Index (NFI), the Tucker–Lewis coefficient (TLI), Root Mean Square Error of Approximation (RMSEA), and SRMR (Standardized Root Mean Square Residual) were used. Findings revealed that the fit of the model was good, χ^2^ = 2305.383; DF = 185; TLI = 0.915; NFI = 0.920; RMSEA = 0.077 [0.075–0.080]; CFI = 0.925; Standardized RMR = 0.0450.

Furthermore, parsimony indexes (Parsimony CFI and Parsimony NFI), considering the sample size (*n* = 1979), were also evaluated, balancing the increase of the fit of the model by the inclusion of more free parameters (PCFI = 0.757, PNFI = 0.751), whose values, between 0.60 and 0.80, are indicative of good fit of the model [84,89]. 

Besides, in the validation of a psychometric instrument, any modification of the original version must be confirmed with the AIC (The Akaike Information Criterion) and the MECVI (The Modified Expected Cross-Validation Index) indexes, based on the maximum likelihood method [90,91]. Thus, for the two PCQ models tested, the one with 21 items (without the reverse items) had lower values of MECVI = 1.275 and AIC = 2439.382 than the 24-item model (MECVI = 1.690 and AIC = 3231.712); that is, it presents better external validity and stability in the Lithuanian sample. 

In addition to the quality indexes of the overall model fit, Cronbach’s alpha coefficients were analyzed. The alpha coefficients were higher for the second-order model with 21 items, (α = 0.957) in comparison to the model with 24 items (α = 0.947). Cronbach’s alpha for all first-order factors were satisfactory (self-efficacy α = 0.922; hope α = 0.909; resilience α = 0.796; corrected resilience α = 0.841; and optimism α = 0.737, corrected optimism α = 0.821), indicating a consistent and reliable instrument [88].

For the sake of clarity, the Lithuanian corrected version of the PCQ-24 with 21 items could be termed as the Lith-PCQ-21. To evaluate the reliability and validity of the Lith-PCQ-21 in the CFA, we have calculated Average Variance Extracted (AVE) and Composite reliability (CR). For the Lith-PCQ-21 model, Table 10 shows that CR indices indicate a good reliability for all factors (all above 0.70). In addition, indices of convergent validity indicated no validity concerns, as all factors’ AVE were less than CR and greater than 0.50.

**Figure 8:**
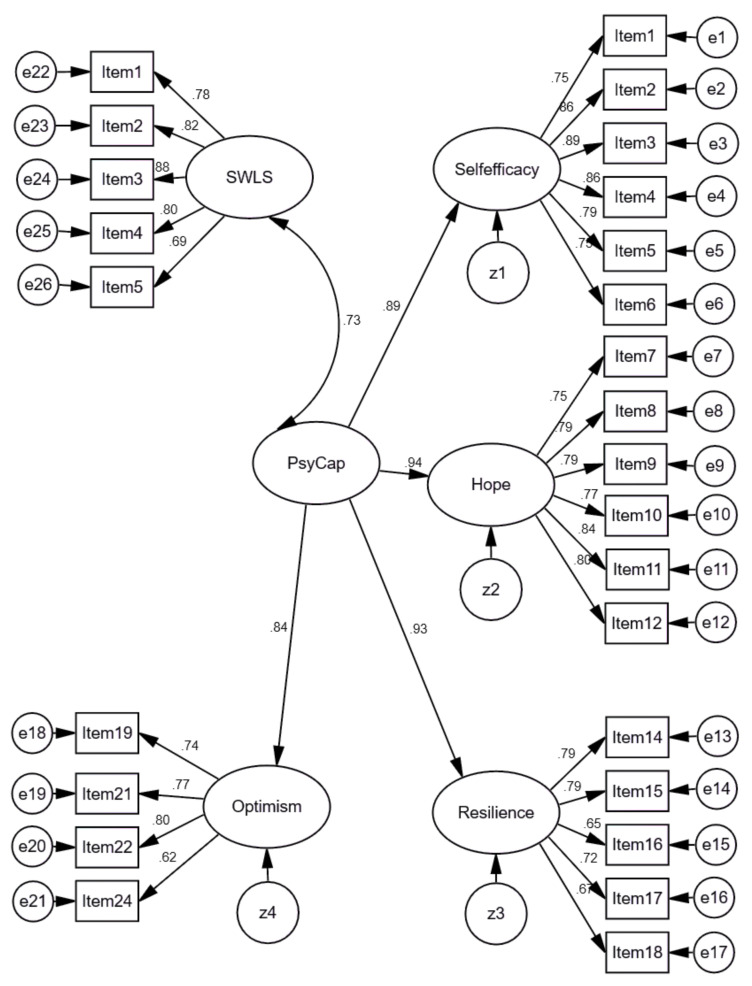
Standardized results of Model 3 on associations between the latent variable of SWLS and the latent variable of PCQ-24 with 21 items (Lith-PCQ-21). χ^2^ = 3088.762; DF = 294; TLI = 0.913; NFI = 0.914; RMSEA = 0.070 [0.068–0.073]; CFI = 0.922; SRMR = 0.0469.

Furthermore, we created a model on associations between the SWLS as a latent variable with five items and the Lith-PCQ-21 as a latent four-factor structure variable with 21 items. Standardized results of the model are presented in Figure 8. In order to assess the model fit, the Comparative Fit Index (CFI), the Normed Fit Index (NFI), the Tucker–Lewis coefficient (TLI), Root Mean Square Error of Approximation (RMSEA), and SRMR (Standardized Root Mean Square Residual) were used. Findings revealed that the fit of the model was good, χ^2^ = 3088.762; DF = 294; TLI = 0.913; NFI = 0.914; RMSEA = 0.070 [0.068–0.073]; CFI = 0.922; Standardized RMR = 0.0469.

The model indicated strong positive associations between a single factor structure of life satisfaction (SWLS) with five items and a second-factor structure of psychological capital (PCQ-24) with 21 items (Lith-PCQ-21). We have chosen to demonstrate this path between a consistent and reliable instrument of SWLS (α = 0.893) and an instrument of PCQ-24 corrected Lithuanian version (Lith-PCQ-21) with 21 items (α = 0.957) because it confirms that both instruments not only have an acceptable validity, including construct validity, but they are also interrelated. 

However, the strong positive associations between psychological capital and satisfaction with life might also suggest that there is a large conceptual overlap between the constructs. Thus, for the sake of completeness, we have tested and compared concurring models: 1. a single-factor model across all PCQ items, 2. a single-factor model across all items across all scales, 3. a two-factor model across all items across all scales, 4. a four- factor model across 24 PCQ items, 5. a four-factor model across 21 PCQ items, 6. a five-factor model across 24 PCQ items. Table 11 displays the FIT indices for the models. Surprisingly, models 4, 5 and 6 demonstrated acceptable fit. As other analyses (the EFA and the CFA) suggested eliminating Items 13, 20 and 23, the four-factor PsyCap 21 model confirms the best fit. Nonetheless, the comparison of the concurring models verified that strong positive associations between the SWLS and PsyCap factors do not indicate conceptual overlap or that the single factor model fits better. 

Furthermore, some previous research indicated gender differences in life satisfaction [77] and psychological capital [78] in different countries. Therefore, an independent-samples T-test was conducted to compare the scores of life satisfaction, self-efficacy, hope, resilience, optimism, and psychological capital (based on Lith-PCQ-21) in groups of the Lithuanian populations’ males and females. No significant differences were found in the scores of satisfaction with life for males (M = 3.8022, SD = 1.21879) and females (M = 3.8002, SD = 1.14454); t(1978) = 0.037, *p* = 0.970. Similarly, there was not a significant difference in the scores of psychological capital for males (M = 4.0903, SD = 0.81352) and females (M = 4.0593, SD = 0.74605); t(1978)= 0.872, *p* = 0.384. Subsequently, there was not a significant difference in the scores of self-efficacy for males (M = 4.1126, SD = 0.98222) and females (M = 4.04024, SD = 0.90499); t(1978)= 1.679, *p* = 0.093; in the scores of hope for males (M = 3.8715, SD = 0.94622) and females (M = 3.7951, SD = 0.92699); t(1978) = 1.783, *p* = 0.075; in the scores of resilience for males (M = 4.0467, SD = 0.86553) and females (M = 4.0241, SD = 0.78069); t(1978) = 0.599, *p* = 0.549, and in the scores of optimism for males (M = 4.3305, SD = 0.81875) and females (M = 4.3776, SD = 0.78926); t(1978) = −1.281, *p* = 0.200. Thus, the research demonstrated no significant differences in the scores of both instruments for female and male groups of the representative Lithuanian sample. 

Furthermore, the SEM analysis of Model 3 has also revealed a good fit for both groups: the male group (χ^2^ = 3088.762; DF = 294; TLI = 0.913; NFI = 0.914; RMSEA= 0.070 [0.068–0.073]; CFI = 0.922), and the female group (χ^2^ = 3088.762; DF = 294; TLI = 0.913; NFI = 0.914; RMSEA = 0.070 [0.068–0.073]; CFI = 0.922). As between groups of females and males, no significant differences, or dissimilarities in responses to the items were found, it can be concluded that the SWLS and the Lith-PCQ-21 might be considered acceptable for applying to monitor life satisfaction and psychological capital of different sexes in the Lithuanian population. 

Additionally, as some previous research suggested the possible impact of rural and urban factors on satisfaction with life [79] and psychological capital [80], we have compared satisfaction with life and psychological capital scores based on the particular living area of respondents (Table 12).

A one-way ANOVA was conducted to compare the effect of living area on life satisfaction and psychological capital. We analyzed the satisfaction with life and psychological capital scores in five groups: rural areas (where live less than 3000 inhabitants), small villages (where live from 3000 to 5000 inhabitants), middle villages (where live from 3000 to 5000 inhabitants), cities (Kaunas, Klaipeda, Panevezys, Siauliai), and the capital of Lithuania, Vilnius (where live around 550,000 inhabitants). An analysis of variance revealed that the effect of living area on life satisfaction was significant, F(4,1975) = 7.626, *p* = 0.000. Similarly, the effect of living area on psychological capital was also significant F(4,1975) = 19.649, *p* = 0.000. A Tukey post hoc test revealed that life satisfaction was significantly higher in the capital (3.9893, ±1.26552) and the biggest cities of Lithuania (3.9219, ±1.14462) in comparison to rural (3.6241, ±1.17700) area (*p* < 0.001). However, there were no significant differences between the scores of life satisfaction in the capital and the biggest cities of Lithuania (*p* = 0.920). Similarly, there were no significant differences in life satisfaction scores between groups of residents living in rural area, small villages, or middle villages. A Tukey post hoc test revealed that psychological capital was also significantly higher in the capital (4.0739, ±0.77858) and the biggest cities of Lithuania (4.2157, ±0.76844) in comparison to rural (3.9215, ±0.72090) area (*p* < 0.001), small (3.4632, ±1.02978) village (*p* < 0.001), or middle (4.0285, ±0.73102) village (*p* < 0.001), but there were no significant differences between the groups in Vilnius and the biggest cities of Lithuania.

Furthermore, some previous research suggested the possible impact of age on satisfaction with life [81] and psychological capital [82]. A correlational analysis, which is displayed in Table 13, demonstrated significant negative correlations between age and psychological capital (r_s_ = −263, *p* < 0.001), including self-efficacy, hope, resilience, and optimism. Similarly, there was found a significant negative correlation between age and life satisfaction, which suggests that older people in Lithuania are less satisfied with life than younger ones.

Additionally, Table 13 also demonstrates strong positive correlations between psychological capital (including its’ dimensions) and satisfaction with life. Similar correlations have been revealed by other researchers [34,36,37,43]. This study should be considered limited as it did not correlate the instruments with other relevant validated Lithuanian scales, but the results indicate that at least these instruments are intercorrelated, which is in line with some other validation studies [68].

## 4. Discussion

This study was the first of its kind to explore in the Lithuanian population the psychometric properties of the SWLS, created by Diener et al. (1985) [10], and the PCQ-24, created by Luthans et al. (2007) [38], which are the leading measures to evaluate satisfaction with life and psychological capital. Though being concerned about the problems of suicides and life satisfaction levels in Lithuania, this study applied a positive psychology framework signifying the importance of accurate evaluation of people’s individual and social resources and psychological wellbeing [24,75]. It was significant to validate the SWLS concerning the fact that Lithuanians’ life satisfaction surveys demonstrated divergent results depending on the assessment tools they used. Moreover, the adaptation of the SWLS and the PCQ-24 in Lithuania is conducive to add to cross-cultural research and benchmarks of well-being research across countries.

### 4.1. Four-Factor PsyCap Structure Confirmed

As mentioned above, the PCQ-24 is based on four published measures on self-efficacy [53], hope [54], optimism [55], and resilience [56]. This research clearly confirmed the existence of a common higher-order PsyCap factor that is superordinate and represents the four sub-dimensions: resilience, self-efficacy, hope, and optimism. The results of exploratory and confirmatory factor analyses verified the psychological capital construct, a latent second-order factor, consisting of the four first-order factors proposed by Luthans et al. (2007) [38]. Like other studies, this research contributed to the robust data on psychological capital, demonstrating that PsyCap is a second-order factor, consisting of the four latent factors: resilience, self-efficacy, hope, and optimism [50,51,52,92,93].

### 4.2. Poor Fit of Negatively Worded Items Identified

Even though in our research the latent variable of PsyCap was found to consist of four latent variables of self-efficacy, hope, optimism, and resilience, but the EFA and the CFA analyses of the PCQ-24 findings in the Lithuanian population showed some points of poor fit of negatively worded items. In this research, the three (negative) inverted items (13, 20 and 23) had factor loads below the minimum values required for exploratory and confirmatory factor analyzes [88]. Some research has also indicated that negatively worded items tend to be problematic when measuring positive constructs [71], and, specifically, psychological capital [62,72,73], and several studies indicated that the reversed items’ exclusion increased the factor load of the other items and increased the model fit [69,94,95,96]. 

### 4.3. Negatively Worded Items Excluded from the Lithuanian Version of the PCQ

Even though the literature provides several tips on how to deal with effects arising from the negative item wording [67,68], it was decided to exclude items 13, 20 and 23 from the Lithuanian version of the PCQ instrument. As mentioned above, several researchers suggested excluding negatively worded items (13, 20 and 23) from the final solutions [69] to increase the model fit. Thus, the results obtained in the Lithuanian sample are similar to those obtained in the Brazilian sample [69], the Turkish sample [94], the Chinese sample [95], and the Portuguese sample [96], among others. 

### 4.4. The Lith-PCQ-21 Reliability and Validity Confirmed

Based on the Lithuanian population results, we opted for the second-order model of 21 items that presented the best fit supported by CFI, TLI, NFI, RMSEA, SRMR, AVE, CR, and DV. Furthermore, for the two PCQ models tested with the AIC and the MECVI indexes [90,91], the one with 21 items demonstrated better external validity and stability in the Lithuanian sample. In addition, the alpha coefficients indicated a consistent and reliable instrument [88]. Like other validation studies [66,67,68,69,70], this research provided some evidence that psychometric indicators of the psychological capital questionnaire demonstrate the precision of the model with reasonable indexes, the reliability of the factorial structure, and the instrument’s internal consistency. As in some other studies [68], this research not only supported previous theoretical considerations that PsyCap is a core construct but confirmed the model of Luthans et al. (2007) [38], evidencing the instrument as an adequate measure to evaluate psychological capital in the Lithuanian population. Yet, as mentioned above, this study provided only limited insights on the validity of the scale.

### 4.5. The Lithuanian Version of the SWLS Validated

During the last decades, numerous studies have documented that the construct of life satisfaction can be reliably measured using the SWLS [1,10,11,12,92,97]. This research provided some valuable psychometric information regarding the construct validity of the Lithuanian version of the SWLS in the Lithuanian population. This research demonstrated the instrument’s internal consistency and supported the propositions of Diener et al. (1985) [10], evidencing the SWLS as an adequate measure to evaluate satisfaction with life. These findings are significant because the Lithuanian population’s life satisfaction surveys demonstrated divergent results depending on the assessment tools they used. Given that there are many suicides in Lithuania and that suicides are negatively related to life satisfaction [15], it was extremely significant to validate reliable instruments that could be used to study the Lithuanian population.

### 4.6. The Associations between Life Satisfaction and PsyCap Found

As indicated above, several studies documented that psychological capital is related to life satisfaction [34,36,43] and positive wellbeing [34,39,40,41,42,44], not to mention many positive work-related outcomes [27,33,46,47,48,49]. This research also demonstrated strong positive associations between satisfaction with life and PsyCap. The findings confirmed that both instruments not only have an acceptable validity, including construct validity, but they are also interrelated. The comparison of the concurring models verified that strong positive associations between the SWLS and PsyCap factors do not indicate conceptual overlap. Similar findings have also been demonstrated by other authors [36,37,43]. Therefore, these instruments can be considered appropriate for monitoring the Lithuanian population’s life satisfaction and psychological capital.

### 4.7. Limitations and Future Directions

Several limitations to this study should be noted. First, bias might occur due to the use of self-report measures only and omitting the objective indicators (e.g., socio-economic status, health, etc.). Second, the findings should be regarded with caution having in mind that the respondents were interviewed at their home directly by the interviewers who marked the answers on the questionnaires. Third, even though the researchers raised a goal to analyze the representative sample, but, in fact, the mean age of the sample was a bit higher than the population’s; thus, the generalization should be made with caution. Furthermore, we have considered a narrow set of variables, and it is challenging to locate the SWLS and the PCQ within the nomological network of other validated Lithuanian scales; thus, the claim that the adapted scales are really valid should be made with caution. Furthermore, this study did not completely meet standards for adaptations, suggested by several authors [98,99,100]. Thus, the evidence for validity of the scales in the Lithuanian sample is limited and needs further examination. Finally, the findings on the SWLS and the PCQ-24 in other countries suggest a necessity for longitudinal and comparative studies on the impact of cultural factors, considering the more specific aspects of each culture.

### 4.8. Theoretical Implications

From a theoretical perspective, this study was the first of its kind to explore the psychometric properties of the SWLS and the PCQ-24. The findings confirmed the existence of a common higher-order PsyCap factor that is superordinate and represents the four sub-dimensions: resilience, self-efficacy, hope, and optimism, which was confirmed by other researchers [41,50,52,61,66,67,69,70,92,93]. The research also verified the life satisfaction factor and demonstrated its association with psychological capital, similar to other studies [36,37,43]. Finally, the results confirmed that the Lithuanian version of the PCQ-24 (Lith-PCQ-21) and the SWLS demonstrate acceptable reliability. Analogous results were demonstrated in other validation studies conducted in many countries [1,10,11,12,66,67,68,69,70,97,101,102]. 

However, some theoretical issues demand further investigation. In our opinion, all the negatively worded items of the PCQ-24 (they were strongly correlated in the Lithuanian sample) might reflect the defensive optimism [103], which cannot be a part of the positive psychological resource such as psychological capital. However, this premise needs additional exploration. Next, response sensitivity to negatively worded items was observed in some countries, but not in every country, which indicates the possible impact of cultural or semantic factors. This assumption also needs further consideration.

Furthermore, previous studies on life satisfaction in Lithuania revealed statistically significant correlations of the past negative, the present hedonistic and the future time perspectives with satisfaction with life among adults [104], strong associations between the net income per month and overall satisfaction with life [20], strong associations between life satisfaction and objective and subjective socioeconomic status [21], strong associations between life satisfaction and positive emotional experiences, perceiving life as pleasant, valuable, and meaningful [22], strong associations between life satisfaction, psychological capital, and health [105]. As this study was based on a positive psychology approach focused on exploration about what makes life most worth living [74], one of the implications for future research is identifying the detailed predictors of satisfaction with life and psychological capital, including hope, self-efficacy, resilience, optimism, and creating a longitudinal design for the Lithuanian population.

### 4.9. Practical Implications

From a practical perspective, the results revealed no significant differences, or dissimilarities in responses to the SWLS and the PCQ items between groups of females and males, and it indicates that both instruments might be considered acceptable for applying to monitor life satisfaction and psychological capital of different sexes in the Lithuanian population. These findings are in line with the findings in other countries [69], even though some researchers highlighted gender differences in life satisfaction [77] and psychological capital [78]. 

Additionally, this research indicated the significant effect of living area on life satisfaction and psychological capital. Interestingly, life satisfaction and PsyCap was highest in the capital Vilnius and the biggest cities of Lithuania, and lowest in small Lithuanian villages. We think that these results should be taken into consideration by policymakers. Moreover, research in other countries also suggested the possible impact of rural and urban factors on satisfaction with life [79] and psychological capital [80]. 

Furthermore, this research revealed a significant negative correlation between age and psychological capital and a significant negative correlation between age and life satisfaction, which suggests that older people in Lithuania are less satisfied with life and have less positive psychological resources than younger ones. These results should also be taken into consideration by policymakers. Especially, in regard to research on impact of age on satisfaction with life [81] and psychological capital [82] in other countries.

This study did not address life satisfaction and PsyCap in relation to employment issues. However, several studies on PsyCap could be taken into consideration by Lithuanian policymakers. In 2020, Donaldson et al. provided new evidence that developing PsyCap, based on hope, self-efficacy, resilience, and optimism, could be a robust and inclusive global human resource strategy for enhancing positive functioning at work [52]. Their research showed that PsyCap predicts work adaptivity, proactivity, proficiency, and overall work performance across 15 diverse nations. Even after controlling for age, education level, and gender, PsyCap accounted for most variance across all four measures of work role performance [52]. Some other authors have also found that improving psychological capital can effectively enhance employability [27] which can be important in the Lithuanian context. Moreover, research shows that individuals higher on PsyCap are more likely to engage in opportunities to sustain and improve wellbeing and more likely to persist in efforts to achieve their goals [50]. The capacity to persevere and overcome emotional difficulties may be significant in the Lithuanian context, which evidences the high rates of unsuccessful struggles with emotional burdens. 

Thus, the Lithuanian version of the SWLS and the Lith-PCQ-21 might be considered acceptable for applying to monitor life satisfaction and psychological capital in the Lithuanian population. Furthermore, the instruments could be used to evaluate the effectiveness of the psychological capital interventions or social policy programs aiming to improve psychological wellbeing of the Lithuanian population. 

## 5. Conclusions

This study aimed to explore psychometric properties of satisfaction with life scale (SWLS) and psychological capital questionnaire (PCQ-24) in the Lithuanian population. The EFA and CFA results clearly confirmed the existence of a common higher-order PsyCap factor that is superordinate and represents the four sub-dimensions: resilience, self-efficacy, hope, and optimism. Due to poor fit, three negatively worded items were excluded from the Lithuanian version of the instrument (the Lith-PCQ-21). The psychometric indicators of the Lith-PCQ-21 demonstrated the reliability of the factorial structure, the instrument’s internal consistency, evidencing the instrument as an adequate measure to evaluate psychological capital. Furthermore, this research has also provided some valuable psychometric information regarding the construct validity of the Lithuanian version of the SWLS. The findings demonstrated the instrument’s internal consistency, evidencing the SWLS as an adequate measure to evaluate satisfaction with life. Furthermore, this study confirmed that both instruments (the SWLS and the Lith-PCQ-21) not only have an acceptable reliability and validity, but they are also interrelated. Furthermore, this study provided some information on satisfaction with life and psychological capital, considering sex, age, and living area. Overall, these findings provide some scientific evidence on Lithuanian version of the SWLS and the Lith-PCQ-21 instruments and offers some practical insights for monitoring life satisfaction and psychological capital of the Lithuanian population.

## Figures and Tables

**Table 1:** Pearson correlations of suicides in Lithuania and unemployment, and life satisfaction. Source: Comunale, M. (2020). The persistently high rate of suicide in Lithuania: an updated view [15].

	Suicides in Lithuania	*p*
Unemployment	0.475 **	<0.001
Life satisfaction	−0.644 **	<0.001

** Correlation is significant at the 0.01 level (2-tailed).

**Table 2:** SWLS: descriptive statistics, correlations between the SWLS items, and internal consistency of the scale.

SWLS	Min	Max	Mean	Std. Deviation	Item 1	Item 2	Item 3	Item 4	Cronbach’s Alpha
Item 1	1	7	3.50	1.392	1				0.893
Item 2	1	7	3.70	1.374	0.650 **	1			
Item 3	1	7	4.08	1.363	0.643 **	0.732 **	1		
Item 4	1	7	4.00	1.380	0.579 **	0.609 **	0.705 **	1	
Item 5	1	7	3.64	1.538	0.557 **	0.519 **	0.550 **	0.611 **	

** Correlation is significant at the 0.01 level (2-tailed).

**Table 3:** PsyCap self-efficacy: descriptive statistics, correlations between self-efficacy items, and internal consistency of the sub-scale.

PsyCap Items	Efficacy Subscale	Min	Max	Mean	Std. Deviation	Item 1	Item 2	Item 3	Item 4	Item 5	Cronbach’s Alpha
1	Item 1	1	6	4.18	1.058	1					0.922
2	Item 2	1	6	3.98	1.150	0.649 **	1				
3	Item 3	1	6	3.97	1.151	0.632 **	0.799 **	1			
4	Item 4	1	6	4.05	1.137	0.626 **	0.697 **	0.773 **	1		
5	Item 5	1	6	4.05	1.129	0.537 **	0.704 **	0.681 **	0.662 **	1	
6	Item 6	1	6	4.17	1.073	0.545 **	0.621 **	0.631 **	0.641 **	0.669 **	

** Correlation is significant at the 0.01 level (2-tailed).

**Table 4:** PsyCap hope: descriptive statistics, correlations between hope items, and internal consistency of the sub-scale.

PsyCap Items	Hope Subscale	Min	Max	Mean	Std. Deviation	Item 1	Item 2	Item 3	Item 4	Item 5	Cronbach’s Alpha
7	Item 1	1	6	4.10	1.024	1					0.909
8	Item 2	1	6	3.94	1.240	0.576 **	1				
9	Item 3	1	6	3.77	1.095	0.694 **	0.593 **	1			
10	Item 4	1	6	3.75	1.154	0.520 **	0.586 **	0.564 **	1		
11	Item 5	1	6	3.65	1.120	0.556 **	0.651 **	0.643 **	0.685 **	1	
12	Item 6	1	6	3.71	1.196	0.494 **	0.681 **	0.577 **	0.635 **	0.748 **	

** Correlation is significant at the 0.01 level (2-tailed).

**Table 5:** PsyCap resilience: descriptive statistics, correlations between resilience items, and internal consistency of the sub-scale.

PsyCap Items	Resilience Subscale	Min	Max	Mean	Std. Deviation	Item 1	Item 2	Item 3	Item 4	Item 5	Cronbach’s Alpha	α If Item 1 Is Deleted
13	Item 1	1	6	3.56	1.171	1					0.796	0.841
14	Item 2	1	6	4.16	0.913	0.163 **	1					
15	Item 3	1	6	4.36	1.017	0.186 **	0.679 **	1				
16	Item 4	1	6	3.74	1.146	0.216 **	0.438 **	0.437 **	1			
17	Item 5	1	6	4.13	1.018	0.166 **	0.561 **	0.549 **	0.599 **	1		
18	Item 6	1	6	3.75	1.175	0.144 **	0.467 **	0.531 **	0.450 **	0.487 **		

** Correlation is significant at the 0.01 level (2-tailed).

**Table 6:** PsyCap optimism: descriptive statistics, correlations between optimism items, and internal consistency of the sub-scale.

PsyCap Items	Optimism Subscale	Min	Max	Mean	Std. Deviation	Item 1	Item 2	Item 3	Item 4	Item 5	Cronbach’s Alpha	α If Items 2, 5 Are Deleted
19	Item 1	1	6	4.16	0.986	1					0.737	0.821
20	Item 2	1	6	3.94	1.205	0.155 **	1					
21	Item 3	1	6	4.54	0.918	0.547 **	0.207 **	1				
22	Item 4	1	6	4.29	1.095	0.562 **	0.204 **	0.680 **	1			
23	Item 5	1	6	3.65	1.139	0.213 **	0.376 **	0.200 **	0.257 **	1		
24	Item 6	1	6	4.39	1.026	0.488 **	0.140 **	0.514 **	0.514 **	0.120 **		

** Correlation is significant at the 0.01 level (2-tailed).

**Table 7:** Comparison of the SWLS models with residual covariances and without residual covariances between Items 1 and 2, 3 and 4, 1 and 4: standardized estimates, model fit indices, average variance extracted, composite reliability, and discriminant validity.

			Standardized Estimates	Average Variance Extracted (AVE)	Composite Reliability (CR)	DV	TLI	NFI	CFI
SWLS Model 1 (with residual covariances):				0.764672	0.886089	0.874	0.988	0.997	0.998
SWLS	->	SWLS Item5	0.639						
SWLS	->	SWLS Item4	0.785						
SWLS	->	SWLS Item3	0.906						
SWLS	->	SWLS Item2	0.815						
SWLS	->	SWLS Item1	0.741						
SWLS Model 2 (without residual covariances):				0.7912205	0.89552	0.889	0.944	0.971	0.972
SWLS	->	SWLS Item5	0.69						
SWLS	->	SWLS Item4	0.801						
SWLS	->	SWLS Item3	0.877						
SWLS	->	SWLS Item2	0.824						
SWLS	->	SWLS Item1	0.774						

**Table 8:** Results of Exploratory Factor Analysis for Lithuanian version of the PCQ-24 items: factor loadings based on a maximum likelihood analysis with Varimax rotation with Kaiser Normalization for the 24 items.

	Self-Efficacy	Hope	Optimism	Resilience	Unidentified	Communalities
PsyCap Item3	0.801					0.763
PsyCap Item2	0.750					0.721
PsyCap Item4	0.716					0.715
PsyCap Item5	0.676					0.622
PsyCap Item6	0.601					0.587
PsyCap Item7	0.510					0.647
PsyCap Item1	0.504					0.571
PsyCap Item11		0.746				0.692
PsyCap Item12		0.744				0.660
PsyCap Item10		0.620				0.580
PsyCap Item8		0.611				0.611
PsyCap Item9		0.499				0.621
PsyCap Item22			0.691			0.563
PsyCap Item21			0.686			0.546
PsyCap Item24			0.548			0.348
PsyCap Item19			0.478			0.518
PsyCap Item17				0.606		0.546
PsyCap Item16				0.471		0.467
PsyCap Item14				0.467		0.599
PsyCap Item15				0.442		0.597
PsyCap Item18				0.388		0.451
PsyCap Item23					0.651	0.280
PsyCap Item20					0.595	0.227
PsyCap Item13					0.544	0.225
Eigenvalues	4.511	3.420	2.612	2.280	1.366	
% of variance	18.797	14.249	10.882	9.501	5.691	

**Table 9:** Results of Exploratory Factor Analysis for Lithuanian version of the PCQ items: factor loadings based on a maximum likelihood analysis with Varimax rotation with Kaiser Normalization for the 21 items.

	Self-Efficacy	Hope	Optimism	Resilience	Communalities
PsyCap Item3	0.795				0.762
PsyCap Item2	0.743				0.720
PsyCap Item4	0.696				0.714
PsyCap Item5	0.660				0.621
PsyCap Item6	0.581				0.587
PsyCap Item1	0.488				0.569
PsyCap Item7	0.483				0.646
PsyCap Item11		0.755			0.691
PsyCap Item12		0.749			0.660
PsyCap Item10		0.629			0.574
PsyCap Item8		0.616			0.611
PsyCap Item9		0.507			0.621
PsyCap Item22			0.726		0.557
PsyCap Item21			0.679		0.543
PsyCap Item24			0.548		0.347
PsyCap Item19			0.482		0.517
PsyCap Item17				0.601	0.546
PsyCap Item14				0.504	0.599
PsyCap Item15				0.486	0.597
PsyCap Item16				0.456	0.462
PsyCap Item18				0.407	0.447
Eigenvalues	4.166	3.501	2.725	2.593	
% of variance	19.839	16.673	12.978	12.346	

**Table 10:** The Lith-PCQ-21: Unstandardized and Standardized Coefficients, Average Variance Extracted, Composite Reliability, and Discriminant Validity.

Latent Constructs		Variables	B	SE	β	Average Variance Extracted (AVE)	Composite Reliability (CR)	DV
PsyCap	->	Optimism	0.739	0.032	0.845	0.8145638	0.946064	0.902
PsyCap	->	Resilience	0.946	0.031	0.949			
PsyCap	->	Self-efficacy	1.000		0.898			
PsyCap	->	Hope	0.967	0.033	0.915			
Self-efficacy	->	PsyCap Item6	1.000		0.752	0.671869	0.924387	0.819
Self-efficacy	->	PsyCap Item5	1.106	0.031	0.792			
Self-efficacy	->	PsyCap Item4	1.211	0.030	0.862			
Self-efficacy	->	PsyCap Item3	1.270	0.031	0.891			
Self-efficacy	->	PsyCap Item2	1.231	0.031	0.864			
Self-efficacy	->	PsyCap Item1	0.969	0.029	0.745			
Hope	->	PsyCap Item7	1.000		0.752	0.6261712	0.909404	0.791
Hope	->	PsyCap Item8	1.278	0.036	0.791			
Hope	->	PsyCap Item9	1.126	0.031	0.791			
Hope	->	PsyCap Item10	1.159	0.034	0.768			
Hope	->	PsyCap Item11	1.227	0.032	0.839			
Hope	->	PsyCap Item12	1.254	0.034	0.804			
Resilience	->	PsyCap Item14	1.000		0.791	0.5285982	0.847769	0.727
Resilience	->	PsyCap Item15	1.114	0.030	0.791			
Resilience	->	PsyCap Item16	1.021	0.035	0.646			
Resilience	->	PsyCap Item17	1.018	0.030	0.723			
Resilience	->	PsyCap Item18	1.090	0.036	0.672			
Optimism	->	PsyCap Item24	1.000		0.617	0.5386108	0.822363	0.734
Optimism	->	PsyCap Item22	1.358	0.051	0.792			
Optimism	->	PsyCap Item21	1.109	0.042	0.771			
Optimism	->	PsyCap Item19	1.146	0.045	0.743			

**Table 11:** Comparison of Concurring Models: Model Fit Indices.

Model	χ^2^	df	CFI	TLI	NFI	RMSEA [90% CI]
1. Single-Factor PsyCap 24 Model	5365.562	252	0.827	0.810	0.820	0.103 [0.101–0.105]
2. Single-Factor PsyCap 24, SWLS 5 Model	8810.523	377	0.770	0.752	0.762	0.108 [0.106–0.110]
3. Two-Factors PsyCap 24, SWLS 5 Model	5366.345	298	0.858	0.845	0.851	0.094 [0.092–0.096]
4. Four Factors PsyCap 24 Model	3079.712	248	0.904	0.893	0.897	0.077 [0.075–0.080]
5. Four Factors PsyCap 21 Model	2305.383	185	0.925	0.915	0.920	0.077 [0.075–0.080]
6. Five Factors PsyCap 24 Model (Items 13, 20, 23 as one factor)	2515.326	247	0.923	0.914	0.916	0.069 [0.067–0.072]

**Table 12:** Differences in satisfaction with life and PsyCap based on living area: rural (*n* = 540), small village (*n* = 103), middle village (*n* = 443), biggest cities of Lithuania (*n* = 556), and the capital of Lithuania (*n* = 337).

		N	Mean	Std. Deviation	Std. Error	95% Confidence Interval for Mean	
						Lower B.	Upper B.
Satisfaction with Life	Rural areas (less than 3000)	540	3.6241	1.17700	0.05065	3.5246	3.7236
	Small villages (3000 to 5000)	103	3.4923	1.27473	0.20412	3.0791	3.9055
	Middle villages (5000 to 80,000)	443	3.7494	1.11435	0.05294	3.6454	3.8535
	Cities (Kaunas, Klaipeda, Panevezys, Siauliai)	556	3.9219	1.14462	0.04854	3.8266	4.0173
	Capital (Vilnius, around 550,000)	337	3.9893	1.26552	0.06894	3.8537	4.1249
	Total	1979	3.8011	1.17986	0.02696	3.7483	3.8540
Psychological Capital	Rural areas (less than 3000)	540	3.9215	0.72090	0.03102	3.8605	3.9824
	Small villages (3000 to 5000)	103	3.4632	1.02978	0.16490	3.1294	3.7971
	Middle villages (5000 to 80,000)	443	4.0285	0.73102	0.03473	3.9603	4.0968
	Cities (Kaunas, Klaipeda, Panevezys, Siauliai)	556	4.2157	0.76844	0.03259	4.1517	4.2797
	Capital (Vilnius, around 550,000)	337	4.2146	0.82743	0.04507	4.1260	4.3033
	Total	1979	4.0739	0.77858	0.01779	4.0390	4.1088

**Table 13:** Spearman correlations between life satisfaction, psychological capital, self-efficacy, hope, resilience, optimism, and age.

	Life Satisfaction	PsyCap	Self-Efficacy	Hope	Resilience	Optimism
Life satisfaction	1.000					
PsyCap	0.634 **	1.000				
Self-efficacy	0.560 **	0.908 **	1.000			
Hope	0.657 **	0.914 **	0.773 **	1.000		
Resilience	0.512 **	0.885 **	0.742 **	0.742 **	1.000	
Optimism	0.483 **	0.793 **	0.634 **	0.641 **	0.688 **	1.000
Age	−0.096 **	−0.263 **	−0.213 **	−0.309 **	−0.225 **	−0.163 **

** Correlation is significant at the 0.01 level (2-tailed).

## Data Availability

Data available on request from the corresponding author.
